# Supplementary material for: Direct observation of elemental fluctuation and oxygen octahedral distortion-dependent charge distribution in high entropy oxides
Source: Nat Commun. 2022 Apr 29;13:2358. doi: 10.1038/s41467-022-30018-y (PMC9055071; doi:10.1038/s41467-022-30018-y)
Supplement: Supplementary file 1 — Supplementary Information [file 41467_2022_30018_MOESM1_ESM.pdf]

Supplementary Information for

Direct observation of elemental fluctuation and oxygen octahedral distortion-dependent charge distribution in high entropy oxides

Lei Su[1,#], Huaixun Huyan[1,#], Abhishek Sarkar[2,3], Wenpei Gao[1], Xingxu Yan[1], Christopher Addiego[4], Robert Kruk[3], Horst Hahn[2,3*], Xiaoqing Pan[1,4,5*]

[1]Department of Materials Science and Engineering, University of California, Irvine, CA 92697, USA.
[2]KIT-TUD-Joint Research Laboratory Nanomaterials, Technical University Darmstadt, 64287 Darmstadt, Germany.
[3]Institute of Nanotechnology, Karlsruhe Institute of Technology, 76344 Eggenstein-Leopoldshafen, Germany.
[4]Department of Physics and Astronomy, University of California, Irvine, CA 92697, USA.
[5]Irvine Materials Research Institute, University of California, Irvine, CA 92697, USA.
[#]These authors contributed equally: Lei Su, Huaixun Huyan.
[*]e-mail: horst.hahn@kit.edu; xiaoqinp@uci.edu

This file includes

Supplementary Figures 1-6

Supplementary Table 1

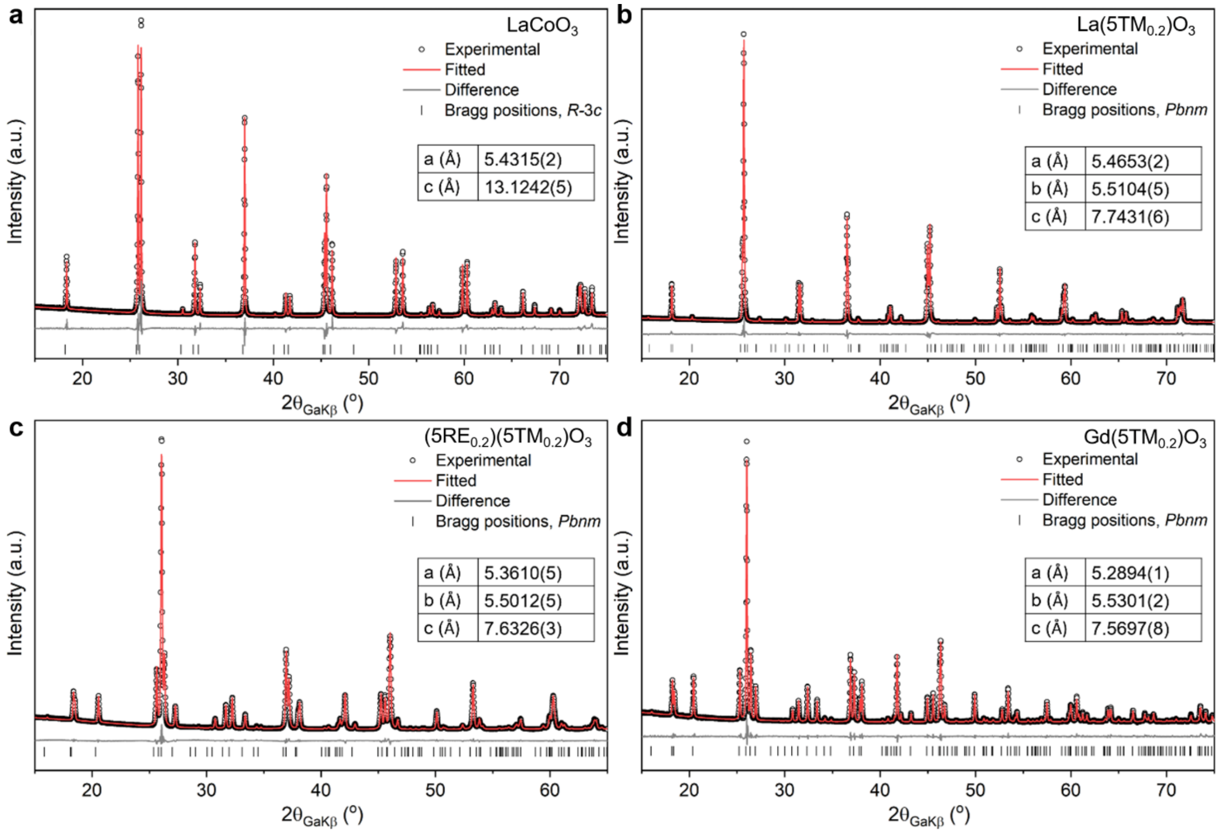

Supplementary Fig. 1. XRD patterns coupled with Rietveld refinements of $LaCoO_3$ and the three P-HEOs. **a** $LaCoO_3$. **b** La(5TM$_{0.2}$)O_3. **c** (5RE$_{0.2}$)(5TM$_{0.2}$)O_3. **d** Gd(5TM$_{0.2}$)O_3.

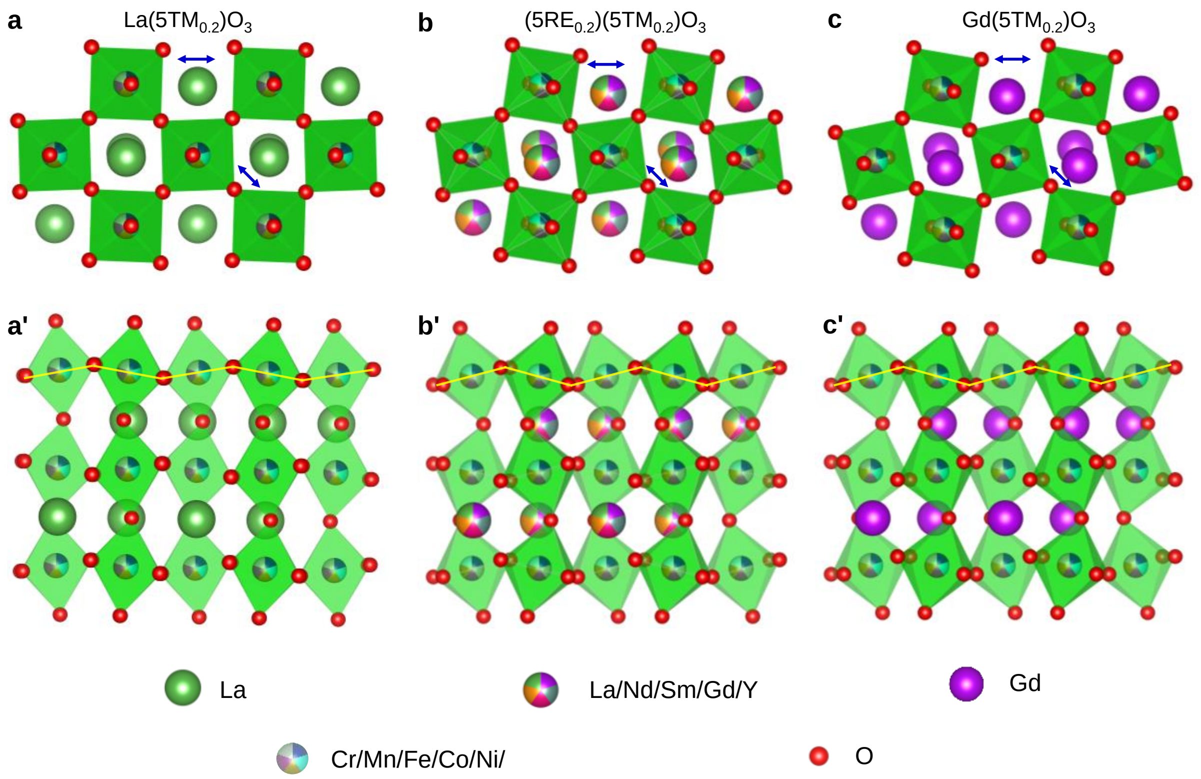

Supplementary Fig. 2. Structure model of the three P-HEOs obtained from the Rietveld refinement of the XRD pattern. **a** to **c** Structure model along [001] projection (view along the *c*-axis), of $La(5TM_{0.2})O_3$, $(5RE_{0.2})(5TM_{0.2})O_3$ and $Gd(5TM_{0.2})O_3$, respectively. **a' to c'** Structure model along [110] projection, of $La(5TM_{0.2})O_3$, $(5RE_{0.2})(5TM_{0.2})O_3$ and $Gd(5TM_{0.2})O_3$, respectively, showing the zig-zag TM-O-TM bond. The blue arrow shows the shift of A-site cations. The yellow line indicates the zig-zag TM-O-TM bond.

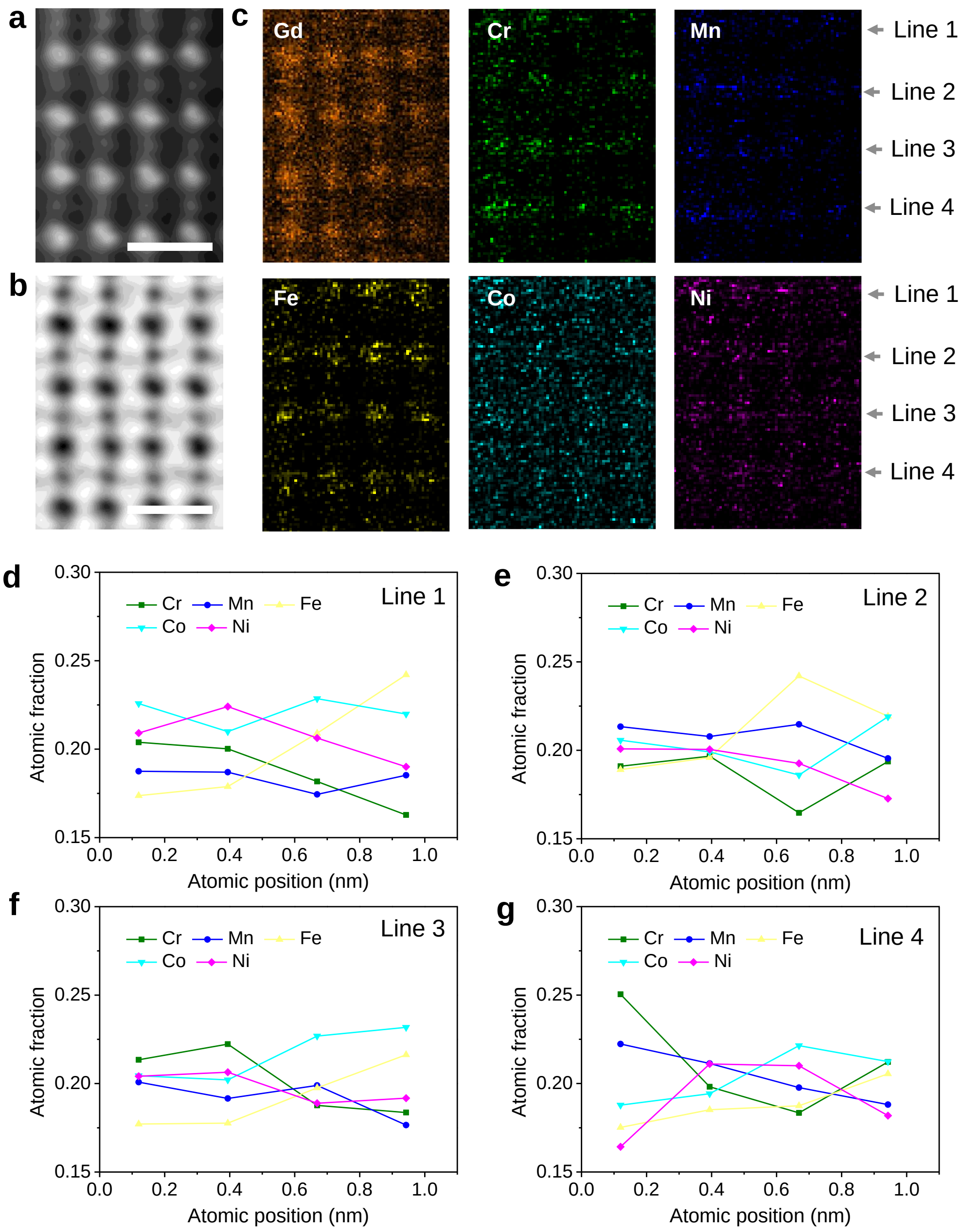

Supplementary Fig. 3. Atomic structure and compositional fluctuation of Gd(5TM$_{0.2}$)O$_3$. **a** HAADF-STEM and **b** ABF of Gd(5TM$_{0.2}$)O$_3$. Scale bar, 0.5 nm. **c** Atomic EDS mapping showing the disordered elemental distribution in the B site of Gd(5TM$_{0.2}$)O$_3$. **d** to **g** Line profiles of the atomic intensity representing the distribution of individual B-site elements in a (001) plane projected along the [110] zone axis.

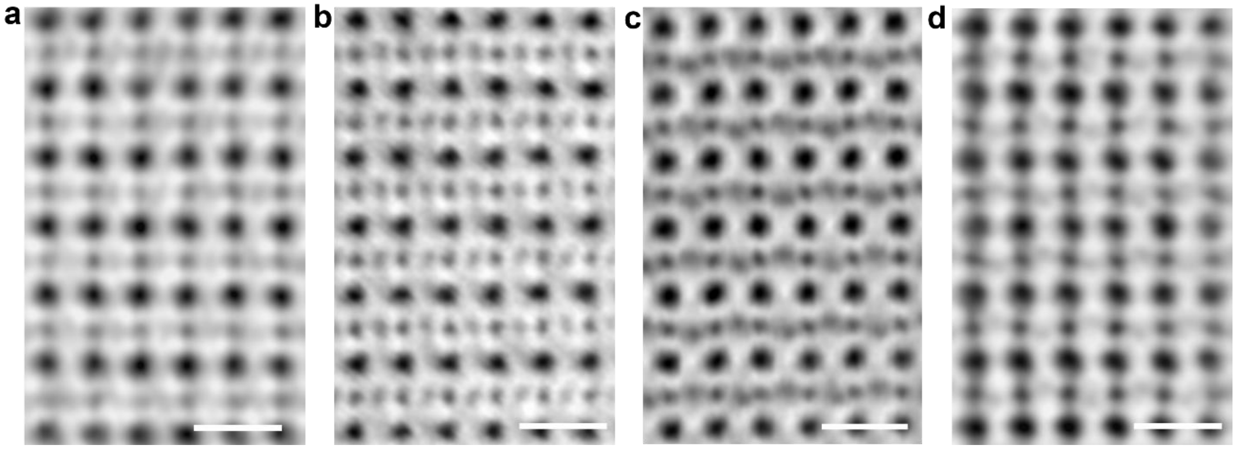

Supplementary Fig. 4. ABF images of $LaCoO_3$ the three P-HEOs. **a** $LaCoO_3$. **b** $La(5TM_{0.2})O_3$. **c** $(5RE_{0.2})$ $(5TM_{0.2})O_3$. **d** $Gd(5TM_{0.2})O_3$. Scale bar, 0.5 nm.

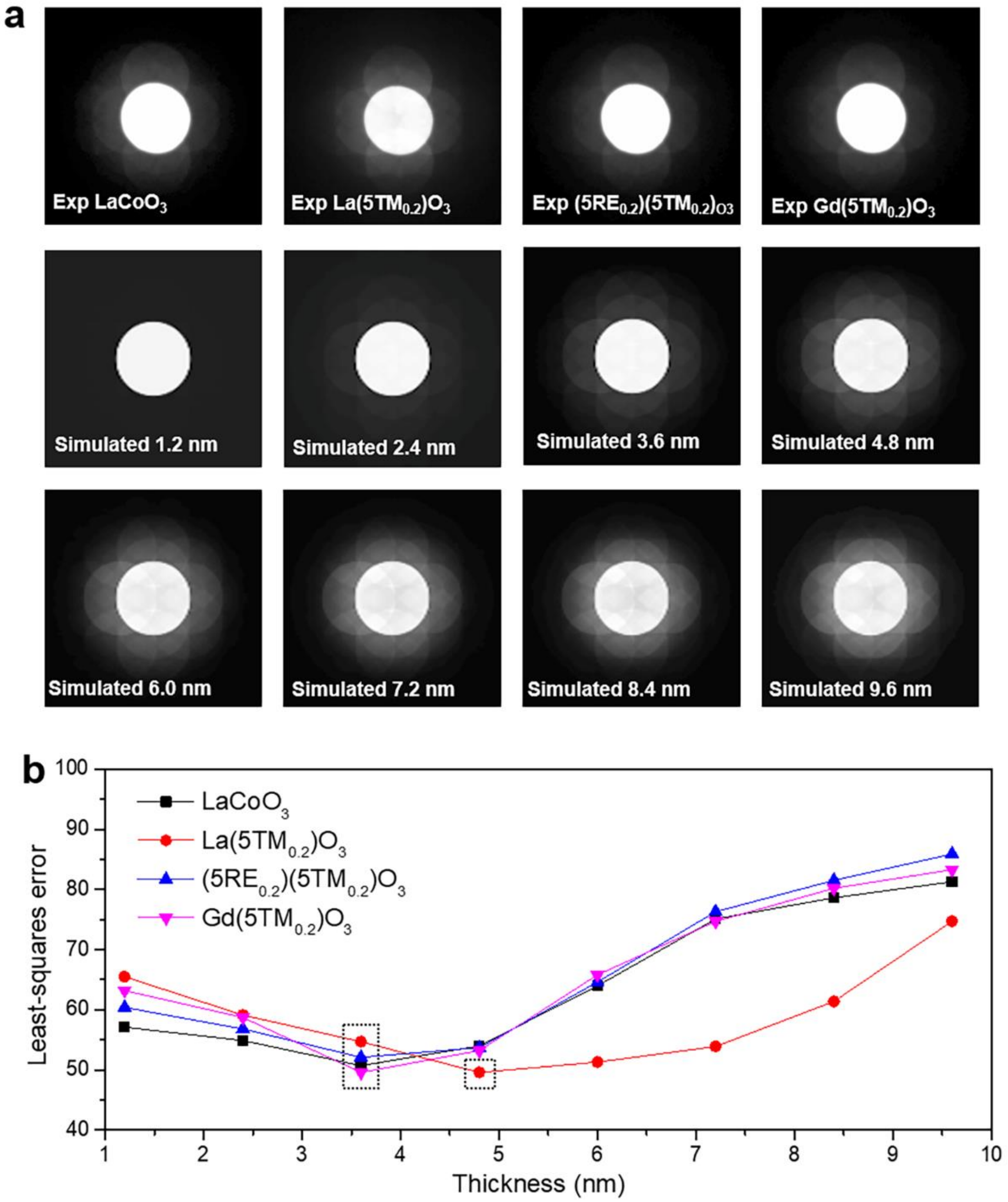

Supplementary Fig. 5. Sample thickness measurement: **a** Experimental PACBED patterns collected from the same regions where the 4D STEM data collected from, and simulated PACBED patterns of $LaCoO_3$ with thickness from 1.2 to 9.6 nm. **b** Least-squares error of the experimental PACBED patterns with the simulated PACBED. The best fit thickness is ~ 3.6 nm for $LaCoO_3$, $(5RE_{0.2})(5TM_{0.2})O_3$ and $Gd(5TM_{0.2})O_3$ and ~ 4.8 nm for $La(5TM_{0.2})O_3$ as highlighted in the black dashed rectangles.

Supplementary Table 1. Statistical data of the TM-O-TM bond angles for $LaCoO_3$ and P-HEOs.

**Samples**	**Distribution of the bond angle, Bin centers (°)**											**Average angle (°)**	**Standard Deviation (°)**
	127.5	132.5	137.5	142.5	147.5	152.5	157.5	162.5	167.5	172.5	177.5		
$LaCoO_3$											30	177.9	1.1
$La(5TM_{0.2})O_3$										11	19	175.6	1.3
$(5RE_{0.2})(5TM_{0.2})O_3$					2	11	6	5	4	1	1	153.4	7.3
$Gd(5TM_{0.2})O_3$	1	4	5	9	6	5						142.6	7.1

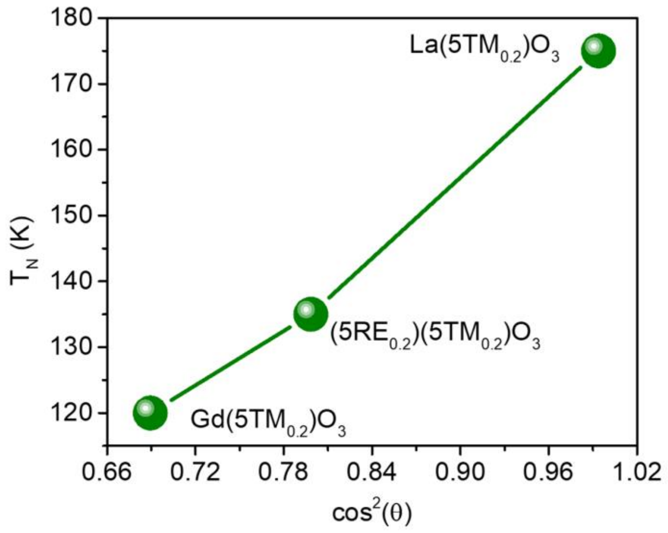

Supplementary Fig. 6. Correlation between the Néel-temperature, T_N, and the average TM-O-TM bond angle θ obtained from the HR-TEM analysis. A lowering of the T_N is observed with deviation of θ from 180º.
